# Design and Implementation of a Low-Complexity Multi-*h* CPM Receiver with Linear Phase Approximation Synchronization Algorithm

**DOI:** 10.3390/e25111530

**Published:** 2023-11-10

**Authors:** Le Wang, Huan Wen, Xucen Wu, Qingping Song

**Affiliations:** 1 School of Information Science and Technology, North China University of Technology, Beijing 100144, China; wh121718@163.com (H.W.); 2021312100115@mail.ncut.edu.cn (X.W.); 2 Beijing Institute of Control and Electronic Technology, Beijing 100038, China

**Keywords:** multi-h CPM, linear phase approximation (LPA), synchronization, FPGA implementation

## Abstract

Multi-*h* continuous phase modulation (CPM), with extremely high spectral efficiency, involves the plague of high demodulation complexity with a large number of matched filters and a complex trellis. In this paper, an efficient all-digital demodulator for multi-*h* continuous phase modulation (CPM) is proposed based on a low-complexity decision-directed synchronization algorithm. Based on the maximum-likelihood estimation of the carrier phase and timing errors, we propose a reduced-complexity timing error detector with linear phase approximation (LPA) to the phase of the multi-*h* CPM. Compared with the traditional synchronization methods, it avoids derivative matched filtering and reduces about 2/3 of matched filters. The estimated accuracy and bit error rate (BER) performance of the LPA-based synchronization algorithm have no loss, as shown by the numerical simulation. Its stability is verified by the derived S-curve. Then, the receivers with the LPA-based synchronization for the three kinds of promising multi-*h* CPM are implemented on a Xilinx Kintex-7 FPGA platform. The experimental results show that the onboard tested BER of the proposed design has an ignorable loss in the numerical simulation. The implementation overhead on FPGA is significantly reduced by about 27% slices, 64% DSPs, and 70% block RAMs compared with the conventional method.

## 1. Introduction

Continuous phase modulation (CPM) is a family of nonlinear modulation schemes with phase continuity and constant envelope. It has been widely used in mobile communication [1], aeronautical telemetry standard [2], industrial communications standards [3], and future satellite broadcasting [4]. It has the advantages of a high-frequency spectrum and power efficiency, as well as a high power of resistance to channel nonlinearity. On the other hand, CPM suffers from the high implementation complexity of synchronization and symbol detection. Multi-*h* CPM, developed with more than one modulation index *h*, has a higher power and spectral efficiency than single-*h* CPM and provides three times the spectral efficiency of PCM/FM [5,6]. Multi-*h* CPM has been selected as the Tier II waveform of the US Advanced Range Telemetry Program Organization (ARTM) [7]. The multiple modulation indices change periodically, which can be coded to improve spectrum efficiency further. Thus, it promises broader application scenarios than the single-*h* CPM. However, more modulation indices bring higher complexity of the trellis and more matched filters, leading to an extremely high detection complexity. Meanwhile, synchronization also has high implementation complexity since the decision-directed algorithm is generally used for the coherent receiver.

The maximum-likelihood sequence detection (MLSD) is used to obtain optimal detection performance for CPM signals. Some decoding algorithms are commonly used to implement the MLSD according to the trellis in the receiver, such as the Viterbi algorithm and the BCJR algorithm [8,9]. It provides significant detection gain compared with the symbol-by-symbol method. However, tremendous complexity is introduced by the trellis of a large number of phase states and branches. Due to its high complexity, some reduced-complexity methods are proposed, such as tilted phase transformation (TPT) [10,11], frequency pulse truncation (FPT) [11,12], pulse amplitude modulation (PAM) [13,14], and state-space partitioning (SSP) [11,15]. All the above methods have been discussed in [16], and have been widely used in various CPM receivers [11,17,18,19,20]. The above algorithms may cause some performance loss in demodulation, except for the TPT method. In practical applications, combining several of them brings a better tradeoff between implementation complexity and performance. These methods also result in a reduction in synchronization complexity. In [21], the decision-directed synchronization for joint phase and timing recovery is introduced with ML estimation for phase and timing error. The synchronization with FPT is also proposed in [22]. The Walsh signal space and PAM decompositions help reduce the synchronization complexity in [23,24,25,26], respectively. In the conventional joint carrier and timing recovery methods mentioned above, the ML estimation of timing error is always calculated by the derivative matched filters because of the nonlinear function of timing offset in the log-likelihood function. It is commonly approached by a finite difference of the outputs from two matched filter banks, of which delays of the on-time MF banks are early and late, respectively, represented as the early–late (EL) synchronizer [27,28]. The EL-based synchronization algorithm is widely utilized for linear [29,30,31] and nonlinear [21,22,26,32] modulation schemes, since it offers high estimation accuracy with low computational cost. The general methods to reduce the complexity of the synchronization depend mainly on the simplification methods of MLSD.

In this paper, we derive a low-complexity synchronization for the multi-*h* CPM. We combine the reduced-complexity MLSD methods of LPA, TPT, and SSP to reduce the number of phase states in the detection trellis as conventional methods. Additionally, we pursue reducing the synchronization complexity of the error signal estimator based on the LPA to the phase of the multi-*h* CPM. With LPA, the timing delay is linearized to the phase. Thus, the derivative MF filters are removed, and the timing error is just estimated by the on-time MF filter banks without using early- and late-MF filter banks. Then, the MF filter banks can be reduced to 1/3 of the original EL-based method. To prove the stability of the proposed synchronizer, we plot the S-curve through theoretical and numerical analysis. Three commonly used multi-*h* CPM schemes, quaternary CPMs with $h=\{\frac{4}{16},\frac{5}{16}\}$, $\left\{\frac{5}{16},\frac{6}{16}\right\}$, and $\left\{\frac{9}{16},\frac{10}{16}\right\}$, are considered to demonstrate the performance of the LPA-based synchronization method. We implement the overall receiver for the above three multi-*h* CPM schemes with LPA-based synchronization and MLSD on a Xilinx Kintex-7 field-programmable gate array (FPGA) platform. The tested bit error rate (BER) results show that the proposed synchronizer’s performance has no loss compared with the conventional EL methods, and the slice and dedicated resource (DSPs and block RAMs) utilization are reduced by about 27% and 67%, respectively. The main contributions of this work are briefly summarized as follows:

- Using LPA, we rederive the ML estimation of phase and timing error signal for multi-*h* CPM, which reduces the complexity of the synchronizer. We modify the LPA-based timing error detector to reduce the complexity further with the sign of the detected symbol. It is friendly to being implemented on FPGA;
- We provide an analytical expression for the S-curve of the proposed error signal detector and analyze its stability through the S-curve;
- We provide an architecture of the receiver with the LPA-based synchronizer and implement it for the three promising multi-*h* CPM schemes on an FPGA platform. The verification results demonstrate a better tradeoff between complexity and performance than the conventional EL-based method.

The structure of this paper is as follows. The signal model and the derivation of the traditional synchronization algorithm and the LPA synchronization algorithm are presented in Section 2. To further reduce the LPA error signal detector, we continue to simplify the detector with the polarization of the error signal. The S-curve is derived to determine the stability of the proposed synchronization algorithm in Section 2. In Section 3, we provide the implementation details based on the receiver’s diagram with the LPA-based synchronization and compare the complexity between the LPA-based algorithm and the conventional EL-based algorithm. Finally, Section 4 illustrates the onboard BER test with the low-complexity receiver for the three multi-*h* CPM schemes to demonstrate the performance of the proposed synchronization algorithm.

## 2. Proposed Synchronization Algorithm Based on LPA

### 2.1. System Model with Tilted Phase Transformation

The general baseband CPM signal [8] is modeled as

(1) $$s\left(t;\alpha \right)=\sqrt{\frac{E_{s}}{T}}e^{j\phi \left(t;\alpha \right)},$$

where *T* is the symbol interval, and $E_{s}$ is the energy per transmitted symbol. The phase of the CPM signal is defined as

(2) $$\phi \left(t;\alpha \right)=2\pi \sum_{i}h_{i}\alpha_{i}q\left(t-iT\right),$$

where $h_{i}=k_{i}/p$ is the modulation index at *i*th symbol interval, while $k_{i}$ and *p* are integers. $h_{i}$ is selected periodically from a set as $\left\{h_{0},h_{1},\ldots \ldots ,h_{N_{h}-1}\right\}$ with a symbol duration, and $N_{h}$ is the number of modulation index. α with $\alpha_{i}\in \left\{\pm 1,\pm 3,\ldots ,\pm \left(M-1\right)\right\}$ is the sequence of *M*-ary information symbols. The phase pulse response q(t) is determined by the *L*-length frequency pulse g(t) as $q\left(t\right)=\int_{-\infty }^{t}g\left(t\right)dt$. Rectangular and raised cosine are commonly used as the frequency pulse shapes with the denotations *L*REC and *L*RC, respectively.

Using TPT, the symbol sequence is mapped to u∈{0,1,…,M−1} with the element $u_{i}=\left(\alpha_{i}+M-1\right)/2$. Then, the number of phase states $N_{state}$ can be reduced from $2pM^{L-1}$ to $pM^{L-1}$ without performance loss [16]. The number of MFs is still $N_{h}M^{L}$. For ARTM CPM as a 4-ary $h=\{\frac{4}{16},\frac{5}{16}\}$ CPM, the trellis using TPT has 256 states and 64 MFs.

We rewrite the phase ϕ(t;α) as

(3) $$\begin{array}{cc} \phi (t;\alpha ) & =2\pi \sum_{i}h_{i}\alpha_{i}q\left(t-iT\right)\\ \; & =\vartheta_{n}+\eta \left(t;l_{n},\alpha_{n}\right),\;nT\leq t<\left(n+1\right)T, \end{array}$$

where $\vartheta_{n}$ is the cumulative phase of the modulator:

(4) $$\vartheta_{n}=\pi \sum_{i=0}^{n-L}h_{i}\alpha_{i}\;\mathrm{mod}\;2\pi .$$

Using TPT, $\vartheta_{n}$ becomes

(5) $$\vartheta_{n}=\theta_{n}+\phi_{n}\;\mathrm{mod}\;2\pi ,$$

where $\theta_{n}$ is the updated cumulative phase:

(6) $$\theta_{n}=2\pi \sum_{i=0}^{n-L}h_{i}u_{i}\;\mathrm{mod}\;2\pi$$

and $\phi_{n}$ is the tilted phase:

(7) $$\phi_{n}=-\left(M-1\right)\pi \sum_{i=0}^{n-L}h_{i}.$$

Note that $\phi_{n}$ is independent of the α or u. Thus, the number of phase states is reduced by half.

$\eta (t;l_{n},\alpha_{n})$ is the correlative phase of the modulator

$$\eta \left(t;l_{n},\alpha_{n}\right)=2\pi \sum_{i=n-L+1}^{n}h_{i}\alpha_{i}q\left(t-iT\right),$$

(8) $$l_{n}=\left[\alpha_{n-L+1},...,\alpha_{n-2},\alpha_{n-1}\right].$$

In Equations (3) and (8), $l_{n}$ is the correlative state vector and $\alpha_{n}$ is the current symbol.

The multi-*h* CPM signal is transmitted over an additive white Gaussian noise (AWGN) channel, and the carrier phase φ and timing offset τ are unknown to the receiver. Hence, the received signal can be modeled as

(9) $$r\left(t\right)=e^{-j\varphi }s\left(t-\tau ;\alpha \right)+w\left(t\right),$$

where w(t) is a complex baseband AWGN with zero mean and single-sided power spectral density $N_{0}$.

### 2.2. Conventional Synchronization Algorithm Based on EL-Matched Filtering

The matched filter (MF) output is

(10) $$Z\left(\tilde{\tau },\alpha_{n}\right)=\int_{(n-1)T}^{nT}r\left(t\right)e^{-j\{\eta \left(t-\tilde{\tau };l_{n},\alpha_{n}\right)+\phi_{n}\}}dt.$$

Assuming that φ and τ are known, the expression of the joint log-likelihood function is [15]

(11) Λ[r(t);φ˜,τ˜]=Re∑n=0N−1e−j(φ˜+θn)Z(τ˜,αn).

The estimated value of the synchronization parameter can be obtained by setting the partial derivative of the likelihood function equal to zero with respect to φ and τ. Thus, we obtain

(12) ∂Λ[r(t);φ˜,τ˜]∂φ=Im∑n=0N−1e−j(φ˜+θn)Z(τ˜,α^n)=0

and

(13) ∂Λ[r(t);φ˜,τ˜]∂τ=Re∑n=0N−1e−j(φ˜+θn)Y(τ˜,α^n)=0,

where $Y(\tilde{\tau },{\hat{\alpha }}_{n})$ is the derivative of $Z(\tilde{\tau },{\hat{\alpha }}_{n})$ with respect to $\tilde{\tau }$. The value of ${\hat{\alpha }}_{n}$ is taken from the best survivor in the Viterbi algorithm or related decoding algorithm.

The carrier phase and timing error signal can be expressed as

(14) eφ(n−D)=Im{Z(τ^n−D,α^n−D)e−j(φ^n−D+θ^n−D)}

and

(15) eτ(n−D)=Re{Y(τ^n−D,α^n−D)e−j(φ^n−D+θ^n−D)}.

The iterative signal expressions of time error and carrier error are as follows

(16) $${\hat{\varphi }}_{n+1}={\hat{\varphi }}_{n}+\gamma_{\varphi }e_{\varphi }\left(n-D\right)$$

and

(17) $${\hat{\tau }}_{n+1}={\hat{\tau }}_{n}+\gamma_{\tau }e_{\tau }\left(n-D\right),$$

where γ is the step size, $\gamma =4BT/k_{p}$, BT is the normalized equivalent noise bandwidth, and $k_{p}$ is derived from the S-curve. *D* is an introduced delay and D=1 produces satisfactory results in many cases (see [21]).

**Figure 1:**
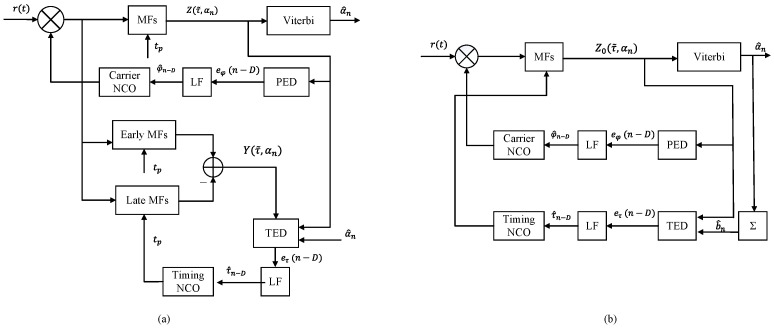
Comparison of synchronization algorithm: (**a**) Schematic diagram of conventional EL-based synchronization algorithm. (**b**) Schematic diagram of LPA-based synchronization algorithm.

The decision-directed (DD) joint phase and timing synchronization, following the error signal detectors of Equations (14) and (15), is shown in Figure 1a. The received signal is synchronized by the estimated ${\hat{\varphi }}_{n}$ and ${\hat{\tau }}_{n}$. The synchronized signal is fed to the on-time-, early-, and late-MF banks. The results of the on-time-MF bank are processed by the Viterbi algorithm (VA). The detected ${\hat{\alpha }}_{n}$ assists in estimating the phase error and timing error signals through a phase error detector (PED) and a timing error detector (TED), respectively. Note that the derivative matched filtering in Equation (15) is implemented by the difference between early- and late-MF banks. Therefore, three MF banks are required: the on-time-, early-, and late-MF banks. Finally, the first-order loop filters update the ${\hat{\varphi }}_{n}$ and ${\hat{\tau }}_{n}$, respectively from Equations (16) and (17). As mentioned in Section 1, such an EL-based timing synchronizer has been widely used in digital receivers for CPM.

### 2.3. Synchronization Algorithm Based on LPA

In order to reduce the complexity of the synchronization besides the phase states trellis, we use the LPA to rederive the synchronization error detector. LPA is a method to approximate the phase response of the CPM as a linear phase response (or a truncated REC phase response), expressed as

(18) $$q_{0}\left(t\right)=\left\{\begin{array}{cc} 0, & t\leq 0\\ \frac{t}{2L^{\prime }T}, & 0<t\leq L^{\prime }T\\ \frac{1}{2}, & t>L^{\prime }T \end{array}\right.$$

where $L^{\prime }$ is the length of the linear phase response. Note that $L^{\prime }$ also stands for the truncated length of PT. LPA is similar to the PT method, and we use PT as one of the reduced-complexity methods for MLSD. Therefore, the number of phase states in the trellis is reduced to $pM^{L^{\prime }-1}$. For a 4-ary $h=\{\frac{4}{16},\frac{5}{16}\}$ CPM, $N_{state}$ becomes $pM^{L^{\prime }-1}=64$, and the number of MFs becomes $N_{h}M^{L^{\prime }}=32$. The signal s(t,α) using TPT and LPA can be rewritten as

(19) $$s_{0}\left(t,\alpha \right)=\sqrt{\frac{E_{s}}{T}}e^{j\phi_{0}\left(t,\alpha \right)}$$

with

(20) $$\phi_{0}\left(t,\alpha \right)=\theta_{n}^{\prime }+\phi_{n}+\eta_{0}\left(t;{l^{\prime }}_{n},\alpha_{n}\right),$$

where

(21) $$\theta_{n}^{\prime }=2\pi \sum_{i=0}^{n-L^{\prime }}h_{i}u_{i}$$

and

$$\eta_{0}\left(t;{l^{\prime }}_{n},\alpha_{n}\right)=2\pi \sum_{i=n-L^{\prime }+1}^{n}h_{i}\alpha_{i}q_{0}\left(t-iT\right),$$

(22) $${l^{\prime }}_{n}=\left[\alpha_{n-L^{\prime }+1},\ldots ,\alpha_{n-2},\alpha_{n-1}\right].$$

Substituting the phase response with Equation (18) into Equation (22), we have the correlative phase with LPA as

(23) $$\begin{array}{cc} \eta_{0}\left(t;{l^{\prime }}_{n},\alpha_{n}\right) & =\pi \sum_{i=n-L^{\prime }+1}^{n}h_{i}\alpha_{i}\frac{t-iT}{L^{\prime }T}\\ \; & =\pi \frac{b_{n}t-c_{n}T}{L^{\prime }T} \end{array},$$

where

(24) $$b_{n}=\sum_{i=n-L^{\prime }+1}^{n}h_{i}\alpha_{i}$$

and

(25) $$c_{n}=\sum_{i=n-L^{\prime }+1}^{n}ih_{i}\alpha_{i}.$$

Note that

(26) $$\phi_{0}\left(t-\tilde{\tau },\alpha \right)=-\frac{\pi b_{n}\tau }{L^{\prime }T}+\phi_{0}\left(t,\alpha \right).$$

Then, the output of the matched filter is

(27) $$\begin{array}{cc} Z_{0}\left(\tilde{\tau },\alpha_{n}\right) & =\int_{(n-1)T}^{nT}r\left(t\right)e^{-j(\eta_{0}\left(t-\tilde{\tau },\alpha \right)+\phi_{n})}dt\\ \; & =\int_{(n-1)T}^{nT}r\left(t\right)e^{-j\left(\pi \frac{b_{n}\left(t-\tilde{\tau }\right)-c_{n}T}{L^{\prime }T}\right)}dt. \end{array}$$

The joint log-likelihood function is

(28) Λ0[r(t);φ˜,τ˜]=Re∑n=0N−1e−jφ˜+θn′+πbnτ˜L′TZ0(τ˜,αn).

The maximum likelihood estimate takes the partial derivative of the phase error and timing error

(29) ∂Λ0[r(t);φ˜,τ˜]∂φ=Im∑n=0N−1e−jφ˜+θn′+πbnτ˜L′TZ0(τ˜,αn)=0

and

(30) ∂Λ0[r(t);φ˜,τ˜]∂τ=Im∑n=0N−1πbnL′Te−jφ˜+θn′+πbnτ˜L′TZ0(τ˜,αn)=0..

Then, the error signal expression is

(31) eφ(n−D)=Im{Z0(τ^n−D,α^n−D)e−j(φ^n−D+θn−D′)}

and

(32) eτ(n−D)=Im{πb^n−DL′TZ0(τ^n−D,α^n−D)e−j(φ^n−D+θn−D′)}=πb^n−DL′Teφ(n−D).

In Equation (32),$\frac{\pi {\hat{b}}_{n-D}}{L^{\prime }T}$ is mainly related to the polarity of the estimated error. To further reduce the complexity of Equation (32), we use the sign of $\frac{\pi {\hat{b}}_{n-D}}{L^{\prime }T}$ instead of itself, and Equation (32) becomes

(33) $$\begin{array}{cc} e_{\tau }^{s}\left(n-D\right) & =\mathrm{sign}\left(\frac{\pi {\hat{b}}_{n-D}}{L^{\prime }T}\right)e_{\varphi }\left(n-D\right)\\ \; & =\mathrm{sign}\left({\hat{b}}_{n-D}\right)e_{\varphi }\left(n-D\right). \end{array}$$

sign(x) is the function of extracting the sign of *x* as

(34) $$\mathrm{sign}\left(x\right)=\left\{\begin{array}{cc} 1, & x>0\\ 0, & x=0\\ -1, & x<0 \end{array}\right..$$

Equation (33) can be implemented on the FPGA platform more efficiently than that of Equation (32). The iterative signal expressions of time error and carrier error are as follows:

(35) $${\hat{\varphi }}_{n+1}={\hat{\varphi }}_{n}+\gamma_{\varphi }e_{\varphi }\left(n-D\right)$$

and

(36) $${\hat{\tau }}_{n+1}={\hat{\tau }}_{n}+\gamma_{\tau }e_{\tau }^{s}\left(n-D\right).$$

### 2.4. Comparison between EL-Based and LPA-Based Synchronization Algorithms

It can be seen from the comparison between the two timing error formulas of Equations (15) and (32) that the proposed timing error detector omits the derivative operation. The modified synchronization algorithm with LPA is constructed in Figure 1b. Compared with the EL synchronization algorithm shown in Figure 1a, the EL MF banks are saved, and the amount of MFs is reduced by 2/3. Based on the TPT, FPT with $L^{\prime }=2$, and SSP with $p^{\prime }$-value phase state partition ($p^{\prime }=4$), the trellis state number $N_{state}$ is decreased from 512 to $p^{\prime }M^{L^{\prime }-1}=16$, and the specific complexity comparison of the above two synchronizers for the three multi-*h* CPM schemes is provided in Table 1. Note that EL-based synchronizer requires 3 MF banks for on-time-, early-, and late-MF paths with $3N_{h}M^{L^{\prime }}=96$ MFs. Here, $L^{\prime }=2$ brings lower performance loss compared with $L^{\prime }=1$ [16]. Thus, the $L^{\prime }=2$ is also set for LPA-based synchronization. Table 1 shows that under the same 16-state trellis, the LPA-based estimator from Equation (32) requires no subtractor and only 1/3 MFs of the EL-based method due to the reduction in the derivative in Equation (15). Note that the LPA-based estimator of the timing error signal, with $MN_{state}=64$ branches of the trellis, has 64 multipliers more than the EL-based estimator. To avoid those multipliers usage, we propose the simplified LPA (SLPA) estimator from Equation (33) with the sign of the estimated ${\hat{b}}_{n}$. It is much simpler than the other two estimators shown in Table 1. With a more complex trellis, the SLPA-based estimator can save more MFs and multipliers.

### 2.5. S-Curve of the LPA-Based Timing Error Detector

The S-curve is used to identify the stable lock points of the error detector and determine whether any false lock point exists. It is calculated by the mean of the error signals e(n), such as the S-curve for TED

$$S\left(\tau \right)=E(e_{\tau }\left(n\right)|\delta ),$$

where $\delta =\tau -\hat{\tau }$ is the timing offset and E() denotes expectation. The S-curve also evaluates the slope of the S-curve at δ=0 as $k_{p}$. The phase error detector (PED) has no simplification compared with the common method [21]. Thus, we derive the S-curve of the timing error τ based on LPA, expressed as [26]

(37) S(τ)=1N∑n=0N−1πbnL′TImejπbnτL′TZn,

where $Z_{n}$ can be computed as

(38) $$Z_{n}=\int_{(n-1)T}^{nT}e^{j(\eta \left(t;l_{n},\alpha_{n}\right)-\eta^{\prime }\left(t;l_{n}^{\prime },\alpha_{n}\right))}dt.$$

The modulated sequence of $\alpha_{n}$ is generally selected as a long and random vector [26]. To simplify the analysis of the S-curve, we construct the complete set L of the state-vector group {$l_{n}$, $l_{n}^{\prime }$}, and its $N^{\prime }=2M^{L+L^{\prime }-1}$ elements can be enumerated easily. Thus, the S-curve is rewritten as

(39) S(τ)=1N′∑n=0N′−1πbnL′TImejπbnτL′TZn|L).

The results of Equation (39) are calculated by the complete set of the state-vector group instead of a long and random sequence used in Equation (37). Note that the results of Equation (39) are more accurate than those of Equation (37). Hence, $k_{p}$ is obtained by $k_{p}{=dS\left(\tau \right)/d\tau |}_{\tau =0}$, which yields

(40) kp=1N′∑n=0N′−1π2bn2L′2T2Im{jZn|L}.

We also can derive the S-curve for the SLPA detector, given by

(41) SSLPA(τ)=1N′∑n=0N′−1signbnImejπbnτL′TZn|L),

with the slope of SLPA at τ=0

(42) kps=1N′∑n=0N′−1π|bn|L′TIm{jZn|L}.

The $k_{p}^{s}$ values for three quaternary multi-*h* CPM schemes with various modulation indices as $h=\{\frac{4}{16},\frac{5}{16}\}$ $h=\{\frac{5}{16},\frac{6}{16}\}$ and $h=\{\frac{9}{16},\frac{10}{16}\}$ are calculated by Equation (42), as shown in Table 2.

**Figure 2:**
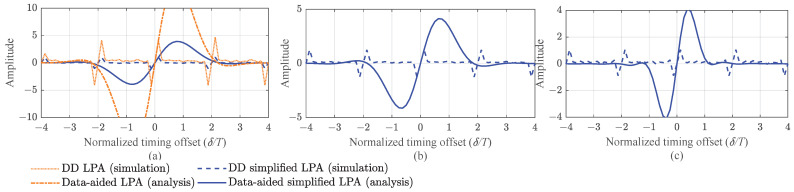
Data-aided (analytic) and decision-directed (simulation) S-curves based on LPA estimator of Equation (32) and SLPA estimator of Equation (33): (**a**) M=4, 3RC, $h=\{\frac{4}{16},\frac{5}{16}\}$. (**b**) M=4, 3RC, $h=\{\frac{5}{16},\frac{6}{16}\}$. (**c**) M=4, 3RC, $h=\{\frac{9}{16},\frac{10}{16}\}$.

Figure 2 presents the S-curves for the decision-directed (DD) and data-aided (DA) timing error detectors based on the LPA of Equation (32) and SLPA of Equation (33). The DD S-curve is simulated by the detector with a random sequence as the transmitted data, and the DA S-curve is calculated by Equation (39). Note that the modulated sequence from set L is known, and such a curve is called the DA S-curve. Three quaternary multi-*h* CPM schemes with various modulation indices as $h=\{\frac{4}{16},\frac{5}{16}\}$, $h=\{\frac{5}{16},\frac{6}{16}\}$, and $h=\{\frac{9}{16},\frac{10}{16}\}$ are considered. The data-aided curves for the two TEDs are computed by Equations (39) and (41), respectively, shown as the solid line in Figure 2. This reveals the correct time at which the timing error detection locks for the three multi-*h* CPM schemes, i.e., τ=0. The dotted lines are decision-directed timing error curves for the three groups of modulation index, which are the mean of the error signal estimation. It can be seen that the decision-directed curves can lock onto the integer periodicity, so the multi-*h* CPM has a stable locking point for the LPA-based method. We also provide the S-curve for the original LPA-based TED for the $h=\{\frac{4}{16},\frac{5}{16}\}$ case. It also has a stable lock point, and the $k_{p}$ of the SLPA-based method is lower than that of the LPA-based method. From the data-aided SLPA S-curves of the three multi-*h* CPM schemes, it can be seen that the S-curves become narrower when the modulation indices increase, which also means higher $k_{p}^{s}$. This is consistent with the results in Table 2.

## 3. System Implementation Based on LPA Synchronization Algorithm

**Figure 3:**
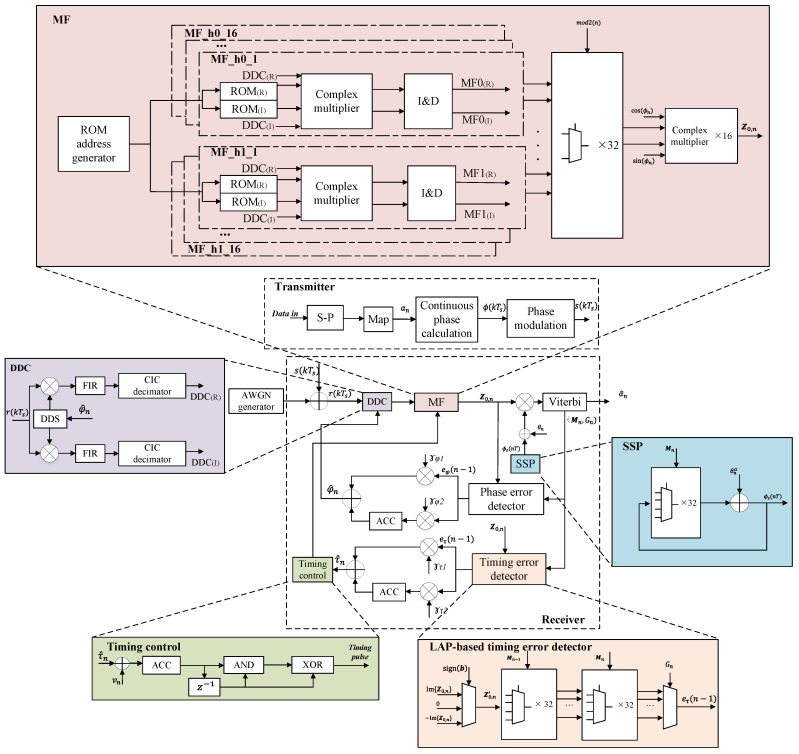
System implementation diagram.

The overall implementation of the multi-*h* CPM system is shown in Figure 3, where the notations are listed in Table 3. Vectors are shown in bold. The subscripts (R) and (I) represent the real and imaginary parts of the outputs, respectively. Using TPT, FPT with $L^{\prime }=2$, and SSP with 4-value phase state partition, the number of the phase state is reduced from 512 for the optimal detection to 16. The LPA-based synchronization removes the usage of EL MF banks to lower the complexity further. With the above reduced-complexity methods, the transmitter and receiver are implemented on Xilinx Kintex-7 FPGA and run at a global clock of 200 MHz and a bit rate of 50 Mbps. The carrier frequency is set to 70 MHz, and the baseband signal is sampled with 8 samples per symbol for the transmitter and receiver. Next, we introduce the implementation details for the main parts of the overall system.

### 3.1. Multi-*h* CPM Transmitter Implementation

In [33], the authors propose a single-*h* and multi-*h* CPM transmitter, which can be reconfigurable with an ignorable increase in memory. It provides a better tradeoff between memory and DSP operations. However, the quantization noise from computing the cumulated phase increases when the modulation indices do not have an exact representation in a given fixed-point format, e.g., $h=\frac{1}{3}$. To deal with this, modular arithmetic units are used to obtain the accurate signal computation for the CPM transmitter in [34]. Here, the modulation indices can be represented accurately in a given fixed-point format. Thus, we use the method based on the read-only memories (ROMs) to calculate the modulated phase (See [33]), which is composed of the correlative phase calculation and cumulative phase calculation. Compared with the integration-based method, the ROM-based method brings lower quantization error and higher complexity. The increased implementation complexity of the modulator can be ignored, considering nowadays software-defined radio platforms. AWGN is generated based on the Box–Muller transform, which provides highly accurate noise samples [35]. The implementation details of the AWGN generator are presented in [36].

### 3.2. Multi-*h* CPM Receiver Implementation

The received signal is sampled and returned to the baseband signal by the digital down conversion (DDC) module. The baseband signal is fed to the MF banks, and the outputs are used for the Viterbi detector. The Viterbi algorithm detects the transmitted sequence and provides information about the surviving path index vector $M_{n}$ and global winning state index $G_{n}$. The proposed synchronization algorithm estimates the phase and timing error signals ($e_{\varphi }\left(n\right),e_{\tau }\left(n\right)$) from the PED and TED utilizing the same matched filter. The second-order loop filters are implemented to update the estimated timing and carrier phase. Finally, the synchronized local carrier signal is feedback to DDC. Some of the primary blocks of the receiver are considered, and efficient implementations of these blocks are described. Optimization details are discussed to achieve high throughput, as follows.

#### 3.2.1. Digital Down Conversion (DDC)

The DDC unit is used to move the received intermediate frequency (IF) signal to the baseband and consists of a direct digital synthesis (DDS), a mixer, and two consecutive filters. DDS outputs the synchronized carrier at IF 70 MHz, which is updated by the estimated carrier phase ${\hat{\varphi }}_{n}$. The mixed complex signal is filtered by two low-pass FIR filters to eliminate the noise and interruption.

#### 3.2.2. Matched Filter (MF) Banks

The MF banks are detailed in Figure 3. It can be seen that a total of 32 MFs are required for the odd- and even-interval modulation indices using reduced-complexity methods, such as TPT, FPT with $L^{\prime }=2$, and SSP with 4-value phase state partition. Each MF unit is built referring to Equation (27). The ROM stores the MF coefficients $e^{-j(\eta_{0}\left(t,\tilde{\alpha }\right)+\phi_{n})}$ for $\tilde{\alpha }=\left\{{\tilde{\alpha }}_{n-1},{\tilde{\alpha }}_{n}\right\}$, and the integral is implemented in discrete time by the complex multiplier and the integrate and dump (I and D) filter. Compared with the FIR-based MF, it reduces the usage of complex multipliers and adders and brings higher throughput. The MF outputs are selected according to the trellis with the odd- and even-symbol intervals and are corrected by the tilted phase.

#### 3.2.3. State-Space Partitioning (SSP) Unit

The SSP algorithm is a decision feedback scheme that could reduce the trellis state according to the partitioning maps. We partition the cumulative phase states of the original trellis from p=16 into $p^{\prime }=4$. Thus, the branch metrics calculated by the MF banks have to be compensated by the estimated surviving phase ${\hat{\phi }}_{s}\left(nT\right)$, which is obtained from the previous surviving phase ${\hat{\phi }}_{t}\left((n-1)T\right)$ and the modified partitioned phase $\theta_{n}^{\alpha }=2\pi h_{n}{\hat{\alpha }}_{n}$ with respect to the estimated ${\hat{\alpha }}_{n}$.

#### 3.2.4. Timing Error Detector

TED is implemented by the SLPA method of Equation (33) with the sign of the estimated ${\hat{b}}_{n}$. We use the VA for TED with traceback length D=1 to calculate the timing error signal, of which the inputs are the imaginary part of MF results. Here, the surviving path index vector $M_{n}$ and the global winning state index $G_{n}$ are reused from the VA of sequence detection. The VA for TED is implemented by two multiplexer banks. Each multiplexer bank is composed of 32 4-to-1 multiplexers. The surviving timing error signal for a phase state is selected through a multiplexer according to the 16-state trellis. The first multiplexer bank calculates the surviving timing error signals using the surviving path index vector $M_{n-1}$ at the (n−1)th symbol interval. These values are transmitted to the second multiplexer bank, and the surviving timing error signals are selected according to the $M_{n}$. Finally, the estimated timing error signal $e_{\tau }\left(n-1\right)$ is selected by the global winning state index $G_{n}$ at *n*th symbol interval due to D=1. Compared with the LPA-TED of Equation (32), it reduces 4×16 real multipliers and reserves the advantage of not using early- and late-MF banks.

#### 3.2.5. Timing Control Unit

The symbol clock is generated using the principle of a numerically controlled oscillator without ROM, which is also similar to DDS. The clock rate is configured by the sum of updated timing error ${\hat{\tau }}_{n}$ and the fixed clock phase increment $v_{n}$. Then, the sum value is accumulated in a fixed word length (set to 32 in general). The highest bit of the accumulator output is the synchronized symbol clock $t_{c}$. Thus, the synchronized timing pulse can be calculated by the following logic expression

(43) $$t_{p}=\left\{t_{c}\left(k\right)⊕t_{c}\left(k-1\right)\right\}t_{c}\left(k\right),$$

where $t_{c}\left(k-1\right)$ is the symbol clock with a sampling interval $T_{s}$ delay.

## 4. Simulation and Analysis

To evaluate the performance of our proposed algorithm, the three commonly used multi-*h* quaternary CPM schemes with $h=\{\frac{4}{16},\frac{5}{16}\}$, $h=\{\frac{5}{16},\frac{6}{16}\}$, and $h=\{\frac{9}{16},\frac{10}{16}\}$ are considered in the following tests. We first analyze the spectrum of the mentioned three CPM schemes. Then, we compare the mean square error (MSE) and BER performances for the CPM with $h=\{\frac{4}{16},\frac{5}{16}\}$ between the proposed LPA-based method and the conventional EL-based method through numerical simulation, denoted as the floating-point simulation. The specifications of the simulation model are based on the implementation requirements as follows:

- Quaternary 3RC CPM, $h=\{\frac{4}{16},\frac{5}{16}\}$;
- IF: 70 MHz;
- Bit rate: 50 Mbps;
- Samples per symbol: 8;
- Carrier offsets: 125 kHz;
- Timing error: 100 ppm.

After performing a floating-point simulation, the receivers with the proposed synchronization algorithm for the three multi-*h* CPM schemes are implemented on a target platform equipped with a Xilinx FPGA Kintex-7 xc7k325tffg900-2. The design is synthesized by the Xilinx synthesis tool (XST). The implementation details are discussed in Section 3. The tested results are presented as the fixed-point simulation.

### 4.1. Power Spectrum Performance

**Figure 4:**
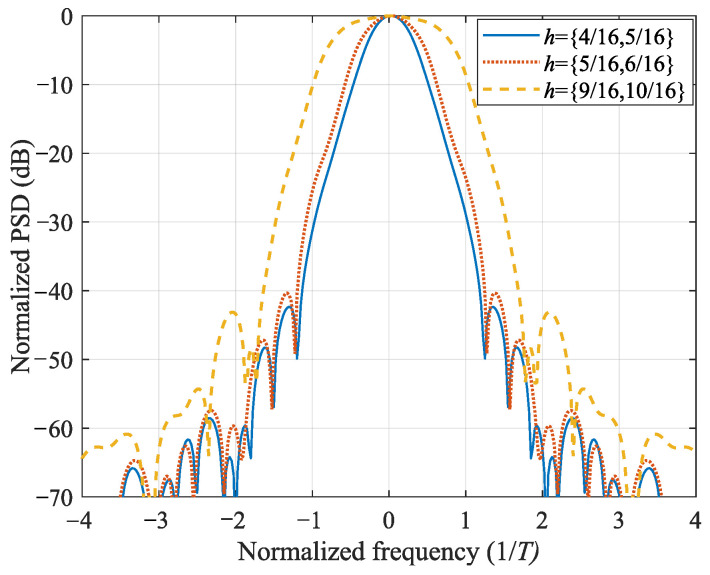
Power spectrum density comparison.

Before the BER performance comparison, we first analyze the power spectrum density (PSD) for the three promising multi-*h* CPM schemes to see their characteristics. The PSD is calculated by the method provided in [8], and the results are shown in Figure 4. We see that the lower modulation index CPM has substantial savings in spectral occupancy. However, it may bring lower minimum square distance, leading to worse BER performance [37]. It is verified in the following test. Note that the three CPM schemes have ignorable difference in implementation complexity using the same reduced-complexity techniques. It seems that the CPM with $h=\{\frac{5}{16},\frac{6}{16}\}$ has a better tradeoff between the spectral efficiency and BER performance than the other two schemes.

### 4.2. MSE and BER Performances Comparison

**Figure 5:**
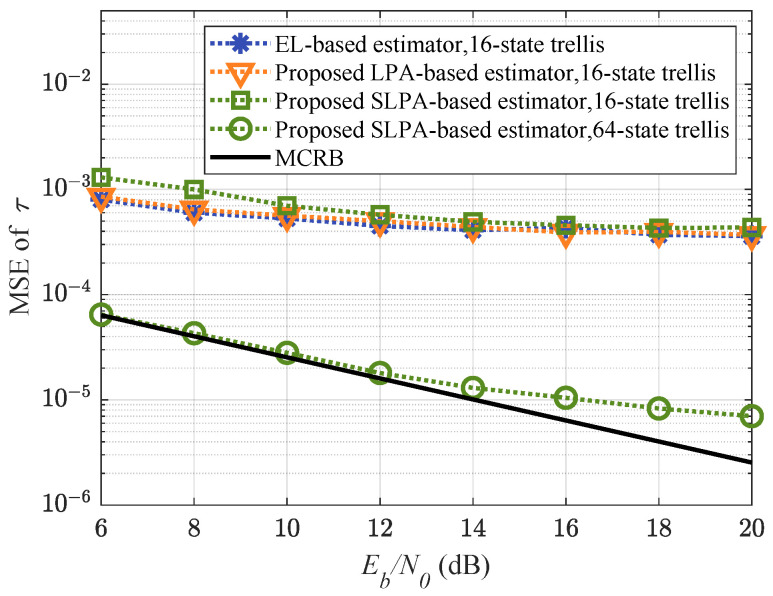
MSE performance comparison.

We next discuss the MSEs of the proposed LPA-based methods. The timing error is estimated by Equation (36) with BT=0.001. We use two reduced-complexity detectors. The first detector has a 16-state trellis, as we implemented in Section 3, with TPT, FPT ($L^{\prime }=2$), and SSP. The second one only uses TPT and FPT ($L^{\prime }=2$) to furhter approach the optimal detector with a 64-state trellis. The results are plotted in Figure 5 and compared with the modified Cramér–Rao bound (MCRB) [26], given by

(44) $$\mathrm{MCRB}\left(\tau \right)=\frac{T^{2}}{8\pi^{2}\bar{h^{2}}C_{a}C_{f}L_{0}}\times \frac{1}{2E_{b}/N_{0}},$$

where $\bar{h^{2}}=1/2\sum_{i=0}^{1}h_{i}^{2}$, $C_{a}=\left(M^{2}-1\right)/3$ for *M*-ary symbols, $C_{f}=3/\left(8L\right)$, and $L_{0}=1/\left(2BT\right)$. Our proposed estimators have ignorable MSE loss compared with the EL-based estimator used in [26,32], since the same reduced-complexity methods are used to simplify the trellis. When the 64-state trellis detector is used, the proposed SLPA-based estimator achieves results close to the performance of MCRB MSE, except for the larger values of $E_{b}/N_{0}$.

**Figure 6:**
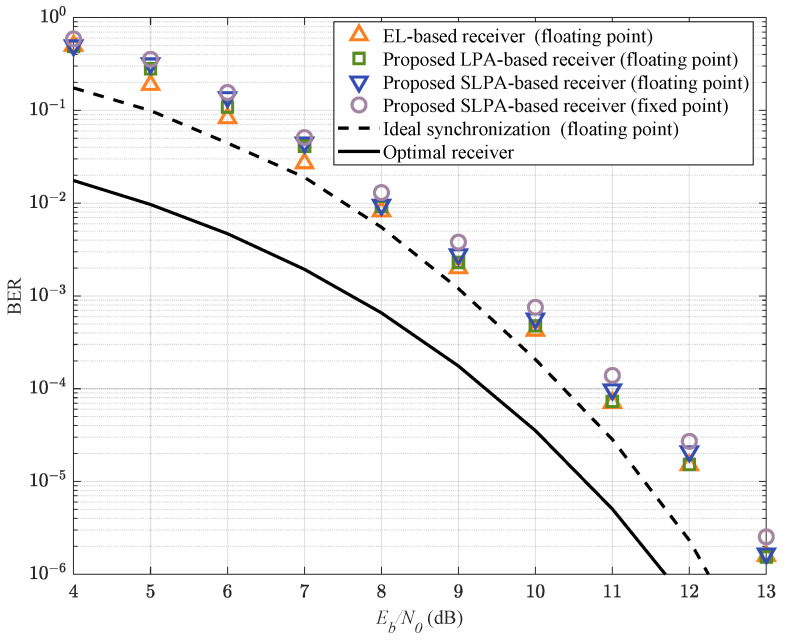
M=4, 3RC, $h=\{\frac{4}{16},\frac{5}{16}\}$ CPM BER performance.

In Figure 6, we compare the BERs for $h=\{\frac{4}{16},\frac{5}{16}\}$ between the proposed methods and EL-based method used in [26,32] under the same trellis (using TPT, FPT with $L^{\prime }=2$, and SSP with 4-value phase state partition). This shows that the LPA-based and EL-based methods have comparable BER performances. However, the LPA-based method has much lower complexity than the conventional EL-based method, which is discussed later. The complexity of the SLPA-based method is lower than that of the LPA-based method, and they have almost the same BER performance. The BERs of the SLPA-based method in floating-point simulation and fixed-point hardware tests are also plotted, and both of them are close to the BER curve of ideal synchronization (without timing and phase error). The theoretical BER bound for the multi-*h* CPM with $h=\{\frac{4}{16},\frac{5}{16}\}$ is calculated by

(45) $$p_{b}=\frac{7}{4^{6}}Q\left(\sqrt{1.288\frac{E_{b}}{N_{0}}}\right)+\frac{\left(4)(648\right)}{4^{6}}Q\left(\sqrt{1.656\frac{E_{b}}{N_{0}}}\right),$$

where

$$Q\left(x\right)=\frac{1}{\sqrt{2\pi }}\int_{x}^{\infty }e^{-u^{2}/2}du.$$

Note that the BER of ideal synchronization has about 0.8 dB degradation to the theoretical BER bound of MLSD at a BER of $10^{-5}$, which is consistent with the conclusion in [16].

**Figure 7:**
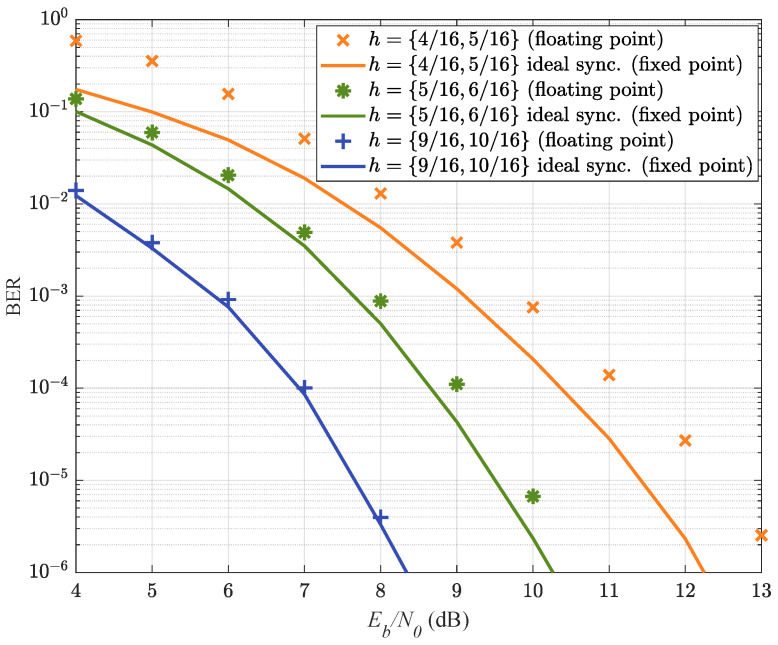
Comparison of the FPGA implementation BER for M=4, 3RC,$h=\{\frac{4}{16},\frac{5}{16}\}$, and $h=\{\frac{5}{16},\frac{6}{16}\}$, $h=\{\frac{9}{16},\frac{10}{16}\}$ CPM schemes.

Next, we show the onboard BER performances of the mentioned three multi-*h* CPM schemes. The LPA-based synchronizer is adopted to reduce the implementation complexity. The system is implemented according to Figure 3. The BERs of the three CPM schemes are plotted in Figure 7. It can be seen that all three CPM schemes have BER performances close to the ideal detection without phase and timing errors. Note that the multi-*h* CPM with higher modulation indices has better BER performance. This is because the higher modulation index brings in a larger minimum squared distance, which promises better BER performance [37]. However, the CPM with lower modulation indices has a higher spectral efficiency, as shown in Figure 4. Thus, among the three tested multi-*h* CPM schemes, the CPM with intermediate-value modulation indices $h=\{\frac{5}{16},\frac{6}{16}\}$ brings better tradeoff between the BER and the spectral efficiency than the other two CPM schemes.

### 4.3. Implementation Complexity Comparison

The overall multi-*h* CPM systems shown in Figure 3 for the three groups’ modulation indices as $h=\{\frac{4}{16},\frac{5}{16}\}$, $h=\{\frac{5}{16},\frac{6}{16}\}$ and $h=\{\frac{9}{16},\frac{10}{16}\}$ are implemented in a platform with the Kintex-7 FPGA. We list the resource usage of the three systems and provide the complexity comparison between the LPA-based receiver and EL-based receiver using TPT, FPT with $L^{\prime }=2$, and SSP with 4-value phase state partition in Table 4.

The transmitters for the three multi-*h* consume almost the same FPGA resources, with data rates of 10 Mbps, 20 Mbps, and 50 Mbps. Under the same 16-state trellis (using the same TPT, FPT, and SSP), the proposed algorithm decreases the 2/3 usage of MFs compared with the conventional EL-based receiver. It brings a reduction in FPGA resources, including slice lookup table (LUT), slice registers, DSPs, and block RAMs. For $h=\{\frac{4}{16},\frac{5}{16}\}$ CPM, compared with the conventional EL-base method, the usages of slice LUT, slice registers, DSPs, and block RAMs are reduced by about 29%, 24%, 64%, and 70%, respectively. It consumes slightly more resources than the other two CPM receivers of $h=\{\frac{5}{16},\frac{6}{16}\}$ and $h=\{\frac{9}{16},\frac{10}{16}\}$ with the proposed synchronizer. Due to the much-simplified structure, it is attractive for the resource constraints tasks and also friendly to implementation and debugging in practical applications.

## 5. Conclusions

In this paper, a low-complexity timing and carrier synchronizer for multi-*h* CPM is proposed based on the LPA method. We derive an ML estimator of the timing and carrier phase error and reduce the derivative MF banks for the TED using LPA. To lower the complexity further, we propose the SLPA-based TED with a sign function replacing multiplication. The S-curve analysis reveals that the proposed LPA-based and SLPA-based TEDs have stable lock points at the correct timing instant. We implement the overall system for the three promising multi-*h* CPM schemes with the SLPA-based synchronizer on FPGA. Compared with the conventional EL-based method, the SLPA-based receivers have ignorable performance loss and save about 27% on average slices and 67% dedicated resources with no loss of BER performance under the numerical simulation and onboard test, which achieves a better tradeoff between the performance and implementation complexity. The proposed synchronization algorithm is studied in the coherent and point-to-point communication system, and the blind estimation will be exploited by the LPA method for burst-mode transmission in the future.

## Figures and Tables

**Table 1:** Complexity comparison of the LPA-based, SLPA-based, and EL-based estimators.

Algorithms	Number of States	Number of MFs	Multiplier	Subtractor
$h=\{\frac{4}{16},\frac{5}{16}\}$ with LPA	16	32	64	0
$h=\{\frac{5}{16},\frac{6}{16}\}$ with LPA	16	32	64	0
$h=\{\frac{9}{16},\frac{10}{16}\}$ with LPA	16	32	64	0
$h=\{\frac{4}{16},\frac{5}{16}\}$ with SLPA	16	32	0	0
$h=\{\frac{4}{16},\frac{5}{16}\}$ with EL in [26,32]	16	96	0	64

**Table 2:** $k_{p}^{s}$ from (42).

CPMs	$h=\{\frac{4}{16},\frac{5}{16}\}$	$h=\{\frac{9}{16},\frac{10}{16}\}$	$h=\{\frac{9}{16},\frac{10}{16}\}$
$k_{p}^{s}$	9.20	11.19	19.04

**Table 3:** Table of symbols.

Symbol	Indication
** $M_{n}$ **	Surviving path index vector for all 16 phase states, and *n* is the symbol index
$G_{n}$	Global winning state index
${\hat{\phi }}_{s}\left(nT\right)$	Surviving partitioned phase
$\theta_{n}^{\alpha }$	Modified partitioned phase with respect to $\alpha_{n}$
$\phi_{t}\left(nT\right)$	Tilted phase
$\gamma_{\varphi 1},\gamma_{\varphi 2}$	Coefficients of the LF for carrier synchronization
$\gamma_{\tau 1},\gamma_{\tau 2}$	Coefficients of the LF for for timing synchronization
${\hat{\varphi }}_{n}$	Estimated carrier phase
${\hat{\tau }}_{n}$	Estimated timing phase
$v_{n}$	Symbol clock phase increment
$r(kT_{s})$	Received signal sampled with $T_{s}$ interval and *k* is the sampling interval index
$s(kT_{s})$	Modulated signal sampled with $T_{s}$ interval
$Z_{0,n}$	MF output vector with the elements calculated by (27) for various branch
sign(b)	Sign vector for various branch calculated by (24) and (34)
$\left\{Z_{0,n}^{\prime }\right\}$	Timing error signal vector with SLPA from (33)

**Table 4:** FPGA implementation complexity comparison.

Transmitter/Receiver		Slice LUTs (203800)	Slice Registers (407600)	DSPs (840)	Block RAMs (445)	Frequency (MHz)	Rate (Mbps)
TX	$h=\{\frac{4}{16},\frac{5}{16}\}$	2472	4073	24	0	300	10,20,50
	$h=\{\frac{5}{16},\frac{6}{16}\}$	2473	4120	24	0	300	10,20,50
	$h=\{\frac{9}{16},\frac{10}{16}\}$	2498	4125	24	0	300	10,20,50
RX	$h=\{\frac{4}{16},\frac{5}{16}\}$, EL in [26,32]	25,352	31,222	1441	118	not specified	not specified
	$h=\{\frac{4}{16},\frac{5}{16}\}$, SLPA	18,065	23,742	520	36	240	50
	$h=\{\frac{5}{16},\frac{6}{16}\}$, SLPA	13,512	17,600	463	36	240	50
	$h=\{\frac{9}{16},\frac{10}{16}\}$, SLPA	14,868	22,220	560	37.5	240	50

## Data Availability

Data are contained within the article.
